# Promising therapeutic potential of tumor suppressor microRNAs for malignant pleural mesothelioma

**DOI:** 10.20517/2394-4722.2022.70

**Published:** 2022-12-05

**Authors:** Shivani Dixit, Agnes Y. Choi, Anand Singh, Karthik Pittala, Nathan Pruett, Chuong D. Hoang

**Affiliations:** Thoracic Surgery Branch, National Cancer Institute, National Institutes of Health, Bethesda, MD 20892, USA.

**Keywords:** Malignant pleural mesothelioma, microRNA, tumor suppressor, therapeutic

## Abstract

Malignant pleural mesothelioma (MPM) is an aggressive and recalcitrant surface neoplasm that defies current multimodality treatments. MicroRNAs (miRNAs) are small noncoding RNAs that epigenetically regulate multiple gene networks and cellular processes. In cancer, miRNA dysregulation is associated with tumorigenesis, with tumor suppressor miRNAs underexpressed or lost, while oncogenic miRNAs are overexpressed. Consequently, miRNAs have emerged as potential therapeutic candidates. Because loss of tumor suppressors predominates the pathophysiology of MPM, re-expressing tumor suppressor miRNAs could be an effective therapeutic strategy. This review highlights the most promising MPM-specific tumor suppressor miRNAs that could be developed into novel therapeutics, the supporting data, and what is known about their molecular mechanism(s).

## INTRODUCTION

MicroRNAs (miRNA) are short noncoding RNAs that post-transcriptionally regulate multiple gene networks and have emerged as potential therapeutic candidates for cancer treatment^[1]^. In principle, miRNAs are less prone to tumor-adaptive resistance or mutations and can exert profound intracellular phenotypic changes upon expression or suppression in cell-specific contexts^[2]^. These properties may be highly relevant to malignant pleural mesothelioma (MPM), a recalcitrant surface cancer that continues to defy therapeutic attempts and is associated with poor survival^[3]^.

According to the World Health Organization classification, MPM is divided into three main histological subtypes: epithelioid (60%−80%), biphasic (10%−15%) and sarcomatoid (10%)^[4]^. Patients with epithelioid histological subtype MPM have a better overall survival (OS) than their sarcomatoid counterparts^[5]^. The OS rates among patients harboring the biphasic subtype depend on the amount of sarcomatoid tumor component. MPM cisplatin-based therapy has not dramatically improved the median survival time of 12 to 18 months^[6]^.

Recently, the USA Food and Drug Administration approved a new therapeutic regimen for MPM patients (primarily for non-epithelioid histology) of combined nivolumab and ipilimumab^[7]^. These drugs target the immune checkpoint inhibitors programmed cell death 1 (PD-1) and cytotoxic T-lymphocyte associated protein 4 (CTLA-4), respectively, and resulted in improved OS compared with standard-of-care chemotherapy (18.1 months *vs*. 14.1 months). Despite these advances, substantial improvements in OS are still elusive, demonstrating the critical need for more effective therapies against MPM.

A PubMed search starting from 2008 and using the terms “microRNA” and “mesothelioma” yielded 204 articles (searched in June 2022). This rough estimate provides context for the selected articles and the total numbers of associated miRNAs. Multiple miRNAs are underexpressed in MPM^[8]^, but only a few have been functionally well characterized, among which several have exhibited potent anticancer activity *in vitro* and *in vivo*. In this review, we highlight MPM-specific tumor suppressor miRNAs in the context of translational potential for clinical use [Table 1]. We apply several criteria to focus on such miRNAs: (1) miRNAs expressed at a low level in human MPM specimens (preferably *vs*. non-cancerous mesothelium); (2) low tumor miRNA expression associated with poor outcomes; and (3) high tumor miRNA expression associated with good outcomes.

## MIRNAS THAT INHIBIT CELL PROLIFERATION AND MIGRATION/INVASION

miR-205 expression is lower in biphasic and sarcomatoid MPM than in the epithelioid type. Ectopic expression of miR-205 in biphasic MPM cells (MSTO-211H) inhibited invasion and cell motility. This was accompanied by downregulation of *ZEB1* and *ZEB2* and upregulation of *CDH1* (E-cadherin), all of which functionally contribute to epithelial-to-mesothelial transition (EMT)^[9]^.

High miR-29c-5p expression in MPM is associated with improved survival irrespective of asbestos exposure^[10]^. miR-29c-5p targets *DNMT1*, *DNMT3A*, and *DNMT3B*, all of which are epigenetic regulators of DNA methylation. Furthermore, overexpression of miR-29c-5p upregulated DNA demethylation genes such as *CTRP1*, *CTRP8,* and Adiponectin. These molecular changes downstream of miR-29c-5p contributed to inhibiting cell proliferation, invasion/migration, and colony formation.

MPM tumor tissues as well as MPM cell lines express low levels of miR-223–3p, which behaves as a tumor suppressor by inhibiting Stathmin (*STMN1*)^[11]^. *STMN1* plays an important role in intracellular transport, mitosis, cell motility, and maintenance of the cytoskeleton. miR-223–3p overexpression in MPM cells reduced *STMN1* levels, which ultimately led to the inhibition of MPM cell proliferation and migration.

Re-expression of miR-1294 suppressed MPM cell proliferation, migration/invasion, and clonogenicity by downregulating *HMGA1*. Interestingly, miR-1294 expression is influenced by the circular RNA (circRNA) polo-like kinase 1 (*circPLK1*) in MPM progression^[12]^. circRNAs are closed noncoding RNAs generated by back-splicing of exons from precursor pre-mRNAs. circRNAs are widely expressed in mammalian cells in tissue-specific patterns and regulate various biological processes. *circPLK1* directly binds miR-1294 by complementary base pairing to sequester its expression in MPM. This reciprocal relationship has been observed in gene profiling studies where *circPLK1* was overexpressed with concomitant miR-1294 downregulation.

## MIRNAS THAT INDUCE CELL CYCLE ARREST AND APOPTOSIS

A microarray analysis of 31 human samples (25 MPM tumors and 6 normal pleura) was performed to identify global miRNA signatures. miR-1–3p was among the top downregulated miRNAs in human MPM tumors compared with normal pleura (6.6-fold)^[13]^. miR-1–3p inhibited MPM cell proliferation and induced G1/S cell cycle arrest and apoptosis. Re-expression of miR-1–3p modulated the expression of signaling molecules that impact cell cycle progression and apoptosis such as *p16, p21*, *p53*, *BAX*, *BCL-2*, and *Survivin*. Another physiologic effect of miR-1–3p was inhibition of cell migration and invasion by downregulating the proto-oncogene *PIM-1*, a serine/threonine-protein kinase^[14]^.

## MIRNAS ASSOCIATED WITH P53

Unlike other solid epithelial tumors, there is a low frequency (approximately 9%) of *p53* mutations in MPM irrespective of histotype^[15]^. However, *p53* functionality is bypassed, wherein 50% to 75% of MPM tumors carry homozygous deletions of *CDKN2A*, which encodes for two cell cycle regulatory proteins in the p53 and RB pathways *i.e*., p16 (INK4a) and p14 (ARF)^[16,17]^. Thus, wild-type *p53* represents a long sought-after therapeutic target in MPM^[18]^. However, leveraging the biologic effects of miRNA could be a more practical approach. Importantly, there are MPM-specific miRNA studies that have described reactivation of *p53* and its physiologic effects. In fact, *p53* regulates the transcriptional expression and biogenesis of a group of miRNAs, concurrently with its gene regulatory network being modulated by miRNAs at multiple levels, *i.e*., a feedback loop^[19]^.

miR-145–5p indirectly regulates *p53* by directly inhibiting its key negative regulator *MDM2*^[19]^. In addition to a potential diagnostic role, miR-145–5p is downregulated in MPM tumors compared with normal pleura due to being silenced by promoter hyper-methylation^[20]^. Furthermore, re-expression of miR-145–5p negatively modulates cell growth, clonogenicity, and migration, while inducing drug resistance and senescence in MPM cells^[21]^. miR-145–5p exerts these antitumor effects in MPM cells by downregulating genes such as *c-MYC*, *OCT4*, and *ZEB1*, all of which mediate core cellular processes. To complement *in vitro* studies, MSTO-211H cells transfected with control or miR-145–5p mimic were subcutaneously implanted into SCID mice to demonstrate differential tumorigenicity. What remains unclear, and a major limitation of this type of *in vivo* model, is whether therapeutic administration of miR-145–5p would result in tumor shrinkage.

miR-215–5p is a unique MPM-associated miRNA that inhibits the malignant features of MPM cells in vitro, largely *via* activating the *MDM2*/*p53* positive feedback loop, which leads to caspase-dependent apoptosis and/or G1/S cell cycle arrest^[22]^. miR-215–5p is downregulated in MPM tumor cells/tissues and is associated with poor prognosis. Ectopic overexpression of miR-215–5p inhibited cell growth and anchorage-dependent and anchorage-independent colony formation in MPM cell lines. The basis for these physiologic effects, aside from *p53* activation, was also partially due to several novel gene targets of miR-215–5p, *i.e*., *CDC7*, *MAD2L1*, *BIRC5*, *LMNB2*, and *RUNX1* (and several not previously associated with MPM). Furthermore, this study verified that in MPM cells, activation of the miR-215/*MDM2*/*p53* positive feedback loop also enhanced the activity of miR-145–5p, another *p53*-inducible miRNA. A single application of miR-215–5p mimic *via* peri-tumoral injection around subcutaneous MPM xenografts in NSG mice was effective at shrinking tumors. Finally, improved survival was observed in mice harboring intra-pleural xenografts (orthotopic) that were treated with miR-215–5p complexed with atelocollagen (*in vivo* delivery vehicle).

The miR-34 family members are *p53*-inducible miRNAs that have anticancer effects in a variety of cancers including MPM^[19]^. The expression of miR-34a-5p, miR-34b-5p, and miR-34c-5p were measured in formalin-fixed, paraffin-embedded (FFPE) MPM tumor samples obtained from patients undergoing extra-pleural pneumonectomy (*n* = 60) and related normal pleura samples (*n* = 23), and the results showed that all were under-expressed (see supplemental data of ref.)^[23]^. *In vitro*, miR-34a-5p was downregulated in MPM subclones that acquired drug resistance with phenotypic rescue upon expression of miR-34a-5p mimic^[24]^. Additionally, transfecting miR-34a-5p mimic into MPM cells reduced cell proliferation, induced cell cycle arrest in G0/G1 phase, and modestly potentiated the anticancer effect of cisplatin^[25]^. MPM tumor cells from Nf2^+/−^/Cdkn2a^+/−^ mice exhibited reduced expression of miR-34a-5p *via* a p53-dependent pathway, which in turn caused elevated *c-MET* expression^[26]^.

Similarly, miR-34b-5p and miR-34c-5p (*i.e*., miR-34b/c) are epigenetically silenced by promoter hypermethylation, accounting for their lowered expression in MPM cells and tissues^[27]^. Ectopic overexpression of miR-34b/c in MPM cells inhibited proliferation and migration/invasion as well as inducing G1/S cell cycle arrest and apoptosis, an effect further enhanced by radiation synergizing with miR-34b/c targeting *BCL-2*^[28]^. The underlying targets that mediate the phenotypic effects of miR-34b/c overexpression include *c-MET*, *c-MYC*, *CDK4/6*, *CCND1*, and *CCNE2*. *In vivo*, an adenovirus vector expressing miR-34b/c intratumorally injected into subcutaneous MPM murine xenografts (average 5 mm diameter) inhibited tumor growth and reduced tumor volumes compared with vehicle^[29]^.

## MIRNAS THAT INHIBIT IMMUNE CHECKPOINTS

Among their multiple molecular targets, some miRNAs affect immune checkpoint regulation, an area of intense basic and clinical research. Interestingly, examples of miRNAs suppressing the immune checkpoint pathway in MPM are contrary to prevailing immuno-oncologic notions. For MPM and other solid tumors, the goal of epigenetic priming for enhanced tumor response to immune checkpoint inhibitors is dependent on the upregulation (not down) of *PD-L1* on tumor cells^[30]^. Furthermore, the hypothetical benefits of downregulating *PD-L1* in MPM (by miRNA regulators as reviewed here) remain unclear, given current clinical trial results. Positive expression of *PD-L1* in MPM is associated with a better objective response rate, and overall survival trended worse with no or loss of *PD-L1* expression^[31,32]^.

The miR-15/16 family of miRNAs (miR-15a-5p, miR-15b-5p, and miR-16–5p) have identical seed sequences, consequently with much overlap in their predicted mRNA targets. All three miRNAs are under-expressed in MPM tumors compared with normal pleura, with miR-16–5p exhibiting the most profound loss and the most significant cell growth inhibition when transfected into MPM cells^[33]^. Re-expression of the miR-16–5p mimic stunted colony formation, induced G0/G1 cell cycle arrest, and increased apoptosis. In the presence of miR-16–5p mimic, there was increased sensitivity to gemcitabine and pemetrexed but not to cisplatin in a panel of MPM cells. These *in vitro* effects correlated with the downregulation of *BCL-2* and *CCND1*. Subcutaneous mouse xenografts were inhibited in a dose-dependent manner with multiple systemic administrations of miR-16–5p mimic loaded into EGFR-targeted bacterially-derived minicells. Another target of miR-16–5p is programmed death-ligand 1 (*PD-L1*), which is downregulated, and is an important factor for immune escape of cancer cells^[34]^. A phase 1, first-in-man trial assessed systemic administration of miR-16–5p mimic to treat MPM and demonstrated a 5% objective response rate (partial response by CT image, *n* = 22) and 68% disease control rate (stable tumor volume by CT image after treatment)^[35]^.

Compared with normal pleura, miR-193a-3p expression is lower in MPM tumors and cell lines as well as being associated with poor overall survival in MPM patients^[36]^. Re-expression of miR-193a-3p mimic in MPM cells inhibited their growth while inducing late apoptosis and necrosis. Some of the underlying affected gene targets associated with the killing of MPM cells included *MCL-1*, which encodes an anti-apoptotic protein of the *BCL-2* family. *In vivo*, miR-193a-3p suppressed tumor growth and induced apoptosis in mouse subcutaneous xenografts. Multiple systemic doses of miR-193a-3p mimic loaded in EGFR-targeted bacterially-derived minicells were required. In a follow-up study, miR-193a-3p modulated *PD-L1* mRNA and protein levels, although in an inconsistent pattern among the panel of tested MPM cell lines^[34]^. Unlike other miRNAs that directly regulate *PD-L1*, miR-193a-3p binds to *PD-L1* transcripts at a noncanonical 8-mer site in the 3′-UTR.

## MIRNAS THAT MODULATE CELL METABOLISM

While the diagnostic utility of miR-126–3p remains to be validated, it was underexpressed in MPM tumors compared with adjacent non-cancerous tissues^[37]^. The *in vitro* effects of miR-126–3p re-expression were assessed in MPM cells under conditions of oxidative stress, mitochondrial dysfunction, mitochondrial DNA depletion, and hypoxia^[38]^. Cell models with ectopic miR-126–3p and stable cell lines expressing miR-126–3p were evaluated. Compared with controls, miR-126–3p reduced glucose uptake, facilitated metabolic reprogramming, and stimulated hypoxia-inducible factor-1α (*HIF-1α*). Primarily, the target that mediated these cellular disturbances downstream of miR-126–3p was insulin receptor substrate-1 (*IRS1*) and its complex signaling network that involves the *Akt/FoxO1/ACL* pathways, converging on *HIF-1α* lowering *VEGF-A* activity (also a direct target of miR-126–3p). These perturbances inhibited MPM cell growth *in vitro*. With similar caveats to the animal model used to test miR-145–5p, MPM cells as subcutaneous xenografts in mice were transfected with miR-126–3p before implantation, but not treated in true *in vivo* fashion upon tumor growth. This study also identified other targets of miR-126–3p, including pyruvate dehydrogenase kinase (*PDK*) and acetyl-CoA-citrate lyase (*ACL*), which induce autophagy when inhibited^[39]^.

## MIRNAS THAT INHIBIT ONCOGENES

Hyperactivated (but non-mutated) tyrosine-kinase-*RAS* signaling is a peculiar pathologic feature of MPM that has been an elusive pathway to target by conventional drugs. miR-206 is a member of the miR-1 family that is downregulated in MPM tissues and cell lines^[40]^. Re-expression of miR-206 inhibited cell growth, clonogenicity, and invasiveness in MPM cell lines. miR-206 caused a G1/S cell cycle arrest and cell death by inducing senescence. miR-206 regulates multiple members of the receptor tyrosine kinase/Ras/cell cycle network (*VEGF-A*, *EGFR*, *c-MET*, and *CDK6*), some of which were also prognostic genes in MPM (*IGF1R*, *KRAS*, *CCND1*, and *CDK4*). Along with *in vitro* anticancer effects, when miR-206 was delivered locally in an anatomic compartment, it suppressed the growth of subcutaneous and orthotopic xenografts, increasing survival in mice. A novel therapeutic strategy consisting of systemic-route abemaciclib (CDK 4/6 inhibitor) and local-route miR-206 was demonstrated to have additive antitumor efficacy, validating the notion that simultaneous knockdown of an oncogenic transcript and inhibition of its protein activity is synergistic.

## AMBIGUOUS TUMOR SUPPRESSOR MIRNAS

Several miRNAs have been purported to be tumor suppressors in MPM largely on the basis of *in vitro* results, but their *bona fide* status in this category remains unclear because of a lack of in-depth studies and characterizations. Using our more stringent criteria for tumor suppressor function, miRNAs whose high expression in MPM tissue was associated with worse prognosis or alternatively whose low expression was associated with better prognosis were excluded from further review except where noted. Furthermore, inconsistency between miRNA expression level and biological activity in MPM cell line experiments suggests that such miRNAs may not be robust candidates for ongoing anti-MPM therapeutic development.

Initially, loss of miR-31–5p was described in MPM cell lines derived from patients with more aggressive disease^[41]^. When re-expressed, miR-31–5p inhibited proliferation, invasion/migration, and clonogenicity of MPM cells. These effects were accompanied by G1/S phase cell cycle arrest, which was attributed to reduced expression of the direct target pro-survival protein phosphatase *PPP6C*. Contrary to these effects, when miR-31–5p was re-expressed in other MPM cells, there was increased chemoresistance, not sensitivity^[42]^. Another discrepancy of miR-31–5p in MPM is that in biphasic and sarcomatoid subtypes (albeit a small sample size from a larger pathologic cohort analysis), its expression was elevated and associated with worse prognosis^[43]^. Therefore, the role of miR-31–5p in MPM remains convoluted.

EphrinA1 binding induced let-7a miRNA, which subsequently repressed the *RAS* proto-oncogene in MPM cells, attenuating proliferation as part of the ephrinA1/EphA2 signaling axis^[44]^. The baseline expression of let-7a in MPM cells was not reported. Furthermore, the relative expression of let-7a in MPM tumors compared with normal tissues remains obscure, with mentions of several different let-7 isoforms varying in their relative expression level in tumors and their importance for either diagnosis or prognosis^[8]^. The primary message in this comprehensive miRNA review was that for let-7 (among several other heavily reported MPM-associated miRNAs), there is no clear consensus of results among heterogeneous studies. A more recent article demonstrated that low let-7c-5p expression, which shares the same seed sequence as all let-7 members and therefore similar targets, in FFPE MPM samples was associated with poor survival^[45]^.

A later study of a similar design seeking to elucidate underlying mechanism(s) of the EphrinA1 axis in MPM implicated miR-302b-3p as a mediator of anti-MPM effects *via* targeting myeloid cell leukemia-1 (*MCL-1*), a member of the anti-apoptotic Bcl-2 family^[46]^. miR-302b levels in MPM are dependent on ephrin A1/EphA2 signaling, whereby administration of Ephrin A1 induced miR-302b expression. Overexpression of miR-302b inhibited cell proliferation, tumorigenicity, and induced apoptotic cell death in MPM cells. This *in vitro* study did not describe the basal expression level of miR-302b-3p in MPM tissues nor any associations with outcomes. A PCR-array miRNA profiling of MPM tumors (*n* = 5 patients) compared with non-MPM tissues identified miR-302b-3p among the top overexpressed miRNAs^[47]^. However, in The Cancer Genome Atlas-meso database, miR-302–3p is nearly undetectable in MPM tumors^[48]^.

The tissue and cell line expression signature of miR-137–3p was heterogenous in both MPM (*n* = 115) and normal pleura (*n* = 23). The variability of miR-137–3p expression, including over- and under-expression, was associated with copy number variation and promoter hypermethylation of miR-137–3p. Regardless of expression levels, miR-137–3p suppressed MPM cell line growth by targeting Y-box binding protein 1 (*YBX1*)^[49]^. Overexpressing miR-137–3p inhibited cell growth and migration/invasion in MPM cells, and unexpectedly even in cell lines with high endogenous expression of the miRNA. An antisense inhibitor of miR-137–3p had no effect on proliferation. Higher miR-137–3p expression was associated with poor, not better, survival among patients who had extra-pleural pneumonectomy or pleurectomy and/or decortication (P/D). These inconsistencies in expression level, biologic activity, and prognosis are difficult to reconcile for this miRNA and more experimentation is required.

## CONCLUSION

The importance of endogenous miRNAs and noncoding regulatory RNAs arranged into complex miRNAs include modulating mRNA signaling networks and diverse aspects of cellular homeostasis. For cancer translational applications, miRNAs with naturally low expression in tumors relative to the normal counterparts represent a practical and promising strategy to further pursue, especially regarding MPM, a recalcitrant surface malignancy without a cure nor durable treatment. This review highlights the surprisingly short list of *bona fide* tumor suppressor miRNAs in MPM with verified gene targets and consistent *in vivo* effects [Table 1]. Thus, there is a continued need to verify the miRNAs already reported but with incomplete pathologic characterization, especially regarding the multiple targets of each miRNA. Doing so will fill in knowledge gaps about the physiology of MPM. Additionally, there is the implied necessity for continued discovery of novel MPM-associated miRNAs, as multiple miRNAs may fulfill an anti-MPM role in the context of inherent inter-tumor molecular and pathologic heterogeneity.

There should be great optimism for miRNA-based therapies for MPM, as novel approaches have addressed the barrier of effective *in vivo* delivery vehicle(s). Notably, the best characterized tumor suppressor miRNAs in this review have been used to demonstrate anti-MPM efficacy. Systemic delivery of miR-16–5p for MPM was well tolerated and safe in a phase 1 setting, as well as showing early signs of activity, warranting additional clinical trials^[35,50]^. More recently, there was successful locoregional (intrapleural and intraperitoneal murine xenograft models) delivery of miR-215–5p and miR-206 complexed as nanoparticles in a composite peptide surface-fill hydrogel (SFH) carrier as a primary and adjuvant MPM therapy^[51]^. SFH is a deformable polymer that can be sprayed onto large anatomically complex surfaces, such as the pleural space, as an adherent coating, which in turn functions as a therapy eluting depot. The encapsulated miRNA nanoparticles that are released from the SFH network can then preferentially enter cancer cells (because of their proximity and cancer-targeting surface properties) to exert anticancer effects. Thus, more reports of miRNAs related to MPM, especially those with tumor suppressor activity, are anticipated.

## Figures and Tables

**Table 1.** Tumor suppressor miRNAs in malignant pleural mesothelioma

			Biological effect		Gene targets		
miRNA	Tissue expression	Prognosis	*In vitro*	*In vivo*	Experimentally validated	*In silico* predicted (number of genes)	Current status
**Inhibit cell proliferation and migration/invasion**							
miR-205^[9]^	Low^#^	-	Inhibits migration/invasion	-	*CDH1, ZEB1, ZEB2*	-	*In vitro*
miR-29c-5p^[10]^	-	High expression = better survival	Inhibits Proliferation Migration/ invasion	-	*DNMT1, DNMT3A, DNMT3B CTRP1, CTRP8,* Adiponectin	*CTRP8*	*In vitro*
miR-223–3p^[11]^	Low	-	Inhibits migration Induces tubulin acetylation	-	*STMN1*	-	*In vitro*
miR-1294^[12]^	Low	-	Inhibits Proliferation Migration/invasion MPM cell stemness	-	*HMGA1*	-	*In vitro*
**Induce cell cycle arrest and apoptosis**							
miR-1–3p^[13,14]^	Low	-	Inhibits Proliferation Migration/invasion Induces G1/S cell cycle arrest Apoptosis	-	*PIM1*	*BDNF, DDX3X, YWHAZ, and 29 total genes common across target databases*	*In vitro*
**Associated with p53 signaling**							
mir-145–5p^[21,22]^	Low	High expression = better survival	Inhibits Proliferation Migration/invasion Drug resistance Induces senescence	Tumor suppression	*c-MYC, MDM2, OCT4*	-	Subcutaneous xenograft*
miR-215–5p^[22,51,52]^	Low	High expression = better survival	Inhibits proliferation Induces G1/S cell cycle arrest Apoptosis Improves Cisplatin efficacy	Tumor suppression	*MDM2, CDC7, MAD2L1, LMNB2, BIRC5, RUNX1, EFNB2*	-	Intrapleural and subcutaneous xenograft
miR-34a-5p^[24,26,53]^	Low	No	Inhibits Proliferation Migration/invasion Drug resistance	-	*c-MET*	-	*In vitro*
miR-34b-5p miR-34c-5p^[27–29,54]^	Low	No	Inhibits Proliferation Migration/invasion Induces G1/S cell cycle arrest Apoptosis Radiosensitivity	Tumor suppression	*c-MET, c-MYC, CDK4, CDK6, CCND1, CCNE2, BCL2, E2F3*	-	Subcutaneous xenograft
**Inhibit immune checkpoints**							
miR-15a-5p^[33]^	Low	No	Inhibits Proliferation	-	*PD-L1*	-	*In vitro*
miR-15b-5p^[24,33,34]^	Low	No	Inhibits Proliferation Drug resistance Induces Apoptosis	-	*PD-L1, BCL-2*	-	*In vitro*
miR-16–5p^[33–35,50,55^]	Low	No	Inhibits Proliferation Clonogenicity Drug resistance Induces G0/G1 cell cycle arrest Apoptosis	Tumor suppression	*BCL-2, CCND1, PD-L1*	-	Subcutaneous xenograft** Phase II clinical trial - planned^†^
miR- L 193a-3p^[34,36]^	Low	High expression = better survival	Inhibits proliferation Induces Apoptosis Necrosis	Tumor suppression	*MCL-1, PD-L1*	-	Subcutaneous xenograft**
**Modulate cell metabolism**							
miR-126–3p[^38,39,56]^	Low	-	Inhibits Mitochondrial function Angiogenesis Induces autophagy	Tumor suppression	*IRS1, VEGF-A, PDK, ACL*	-	Subcutaneous xenograft*
**Inhibit oncogenes**							
miR-206^[40,51]^	Low	-	Inhibits Proliferation Migration/invasion Induces G1/S cell cycle arrest Senescence	Tumor suppression	*KRAS, EGFR, VEGF-A, IGF1R, CDK4, CDK6, CCND1, c-MET*	-	Subcutaneous and intrapleural xenograft

All miRNAs are named according to their sequence identity in miRBase (release 22.1; Available from: www.mirbase.org), which may defer from their nomenclature in the original articles.

# Low in biphasic and sarcomatoid histological subtypes;

* although mouse flank xenografts were reported, the MPM cell line had been transfected beforehand with miR-145–5p and then grafted into the mouse;

** systemic delivery of miRNA;

† as indicated by the reference^[50]^.
